# Effect of Free Treatment and Surveillance on HIV-Infected Persons Who Have Tuberculosis, Taiwan, 1993–2006

**DOI:** 10.3201/eid1502.081000

**Published:** 2009-02

**Authors:** Shu-Hui Tseng, Donald Dah-Shyong Jiang, Hao-Seong Hoi, Hsiu-Yun Lo, Kao-Pin Hwang

**Affiliations:** Centers for Disease Control Department of Health, Taipei, Taiwan (S.-H. Tseng, D.D.-S. Jiang, H.-S. Hoi, H.-Y. Lo); Chang Gung University College of Medicine, Kaohsiung, Taiwan (S.-H. Tseng); Chang Gung Memorial Hospital—Kaohsiung Medical Center, Kaohsiung, Taiwan (K.-P. Hwang)

**Keywords:** HIV/TB coinfection, HAART, surveillance system, mortality, influenza A (H5N1), AZT

## Abstract

In 1997, Taiwan made highly active antiretroviral therapy (HAART) available without cost to HIV-infected persons; in 2001, a national web-based surveillance system was implemented. Healthcare workers use the system to monitor patients' conditions and can intervene when necessary. Free HAART, coupled with the surveillance system, appears to have increased survival rates of HIV-infected persons with tuberculosis in Taiwan.

Most experts believe that complete and efficient surveillance is the top priority in detecting and preventing outbreaks of emerging infectious diseases, such as tuberculosis (TB) and HIV coinfection (*1*–*3*) or influenza virus A (H5N1). In Taiwan, a national web-based surveillance system established in July 2001 provides complete and efficient reporting and management of persons coinfected with HIV and TB and enables healthcare workers to identify noncompliance with therapy and to intervene when necessary. After highly active antiretroviral therapy (HAART) became available free of charge in Taiwan in April 1997, the death rate for HIV-infected persons decreased from 5.7% in 1997 to 1.8% in 2006. To determine whether implementation of the national surveillance system in combination with the availability of free HAART further increased survival rates of HIV-infected persons with TB, we compared their demographic, clinical, and behavioral characteristics during 3 periods: 1) before free HAART was available (1993–1996); 2) after free HAART was available but before the surveillance system was implemented (1998–2000); and 3) after both free HAART and the surveillance system were available (2002–2006).

## The Study

We obtained data on persons with HIV/AIDS and TB from the national databank at the Centers for Disease Control (CDC Taiwan) of the Department of Health, Taiwan. Coinfection with HIV and TB was defined as HIV infection in persons in whom TB was later diagnosed. A total of 660 persons with both HIV and TB were reported during 1993–2006.

We used Microsoft Excel XP spreadsheet (Microsoft, Redmond, WA, USA) and SAS version 9.1 (SAS Institute Inc., Cary, NC, USA) for statistical analysis. The χ^2^ goodness-of-fit test with type I error = 0.05 was used to examine differences in demographic, clinical, and behavioral characteristics of persons with HIV and TB coinfection during 1993–2006. Multivariates for analysis were sex and age, results of sputum smear and sputum culture, pulmonary radiographic diagnosis, TB types (extrapulmonary and nonextrapulmonary), mode of HIV transmission, sexual behavior, compliance with HAART, and use of the surveillance system (Table 1). We used the Kaplan-Meier method (*4*) from SAS to evaluate and compare the effect on survival rates of different factors in persons coinfected with HIV and TB 1 year after reported TB diagnosis.

**Table 1:** Demographic and clinical characteristics of 660 persons coinfected with HIV and TB, Taiwan, 1993–2006*

Characteristics	No. (%) persons	p value†
Sex		<0.0001
M	612 (92.7)	
F	48 (7.3)	
Age, y		<0.0001
<45	498 (75.5)	
>45	162 (24.5)	
Sputum smear (n = 484)		<0.0001
Negative	287 (59.3)	
Positive	197 (40.7)	
Sputum culture (n = 340)		0.0172
Negative	148 (43.5)	
Positive	192 (56.5)	
Pulmonary radiograph results (n = 531)		<0.0001
Normal	54 (10.2)	
Abnormal	477 (89.8)	
Extrapulmonary TB‡		<0.0001
Yes	73 (11.1)	
No	587 (88.9)	
Risk behavior (n = 554)		<0.0001
Sexual	513 (92.6)	
Injection drug user	41 (7.4)	
Sexual behavior (n = 566)		0.0004
Heterosexual	325 (57.4)	
Homosexual or bisexual	241 (42.6)	
Highly active antiretroviral therapy‡ (n = 534)		<0.0001
1998–2006 (free)	493 (92.3)	
1993–1996 (not free)	41 (7.7)	
National web-based surveillance reporting and management system (n = 520)		<0.0001
2002–2006 (available)	386 (74.2)	
1998–2000 (not available)	134 (25.8)	
Outcome§ (n = 606)		<0.0001
Survival	522 (86.1)	
Death	84 (13.9)	

*TB, tuberculosis. †χ^2^ goodness-of-fit test with type I error = 0.05 used to examine differences in demographic, clinical, and behavioral characteristics. ‡Definition following World Health Organization guideline (*1*). §Tracking for 1 year from report of TB diagnosis.

Kaplan-Meier analysis yielded the following results: 63% of persons coinfected with HIV and TB survived during 1993–1996; 78% survived during 1998–2000; and 93% survived during 2002–2006 (p<0.0001) (Figure). We then applied Cox proportional hazards modeling (*5*) to each variable to assess the effect on survival rates after implementation of HAART and the surveillance system. Age <45 years, negative sputum smear, availability of free HAART, and implementation of the national surveillance system substantially increased survival rates of persons coinfected with HIV and TB (Table 2).

**Figure:**
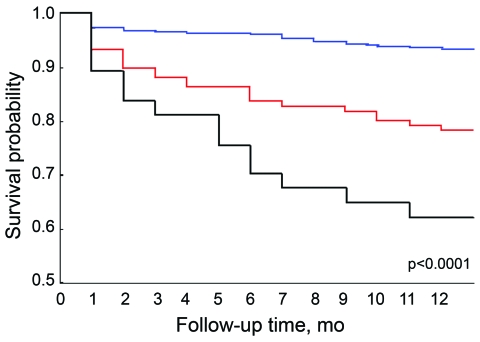
Kaplan-Meier analysis of survival of HIV-infected patients with tuberculosis in Taiwan during 3 different periods: before free highly active antiretroviral therapy (HAART) was available (1993–1996, black line); B) after free HAART was available but before the national web-based reporting and management surveillance system was implemented (1998–2000, red line); and C) after free HAART and the surveillance system were available (2002–2006, blue line).

**Table 2:** Cox regression model of possible risk factors for death among persons coinfected with HIV and tuberculosis, Taiwan, 1993–2006

Risk factor	Hazard ratio	95% Confidence interval	p value
Sex (F)	1.462	0.292–7.303	0.6438
Age (>45 y)	2.907	1.162–7.272	0.0226
Sputum smear positive	2.722	1.008–7.349	0.0482
Pulmonary radiograph abnormal	7.006	0.848–57.916	0.0708
Pulmonary tuberculosis	3.169	0.641–15.674	0.1573
Heterosexual	2.049	0.794–5.290	0.1333
Before availability of free highly active antiretroviral therapy*	8.398	2.170–32.508	0.0021
Before implementation of national web-based surveillance reporting and management system†	7.664	2.115–27.768	0.0019

*1993–1996. †1998–2000.

## Conclusion

Many factors can increase survival rates of HIV-infected persons, such as HAART (*6**–**9*), prevention of opportunistic infections, patient attitude, healthcare worker knowledge, and promotion of health education. Our data indicate that national web-based surveillance reporting and management, coupled with the availability of free HAART, increase survival rates of persons coinfected with HIV and TB (p<0.0001).

Taiwan’s national web-based surveillance system enables healthcare workers to follow, record, and understand the conditions of patients without geographic limitations. Physicians, public health nurses, health administrators, and other healthcare professionals in local through federal government agencies can use the system to follow up and manage the condition of persons coinfected with HIV and TB. For example, public health nurses from national healthcare centers visit such patients regularly, record treatments, and assess their conditions and compliance with therapy; staff from central health department monitor and supervise the condition of each patient through the system. In this way, the system may increase patients’ compliance and thus their survival rates (*10*–*14*).
